# Draft Genome Sequence of a Serratia marcescens Strain (PIC3611) Proficient at Recalcitrant Polysaccharide Utilization

**DOI:** 10.1128/mra.00306-22

**Published:** 2022-07-05

**Authors:** Jessica K. Novak, Jeffrey G. Gardner

**Affiliations:** a Department of Biological Sciences, University of Maryland, Baltimore County, Baltimore, Maryland, USA; University of Southern California

## Abstract

Serratia marcescens is a Gram-negative bacterium found in terrestrial and aquatic environments and studied for its polysaccharide utilization capabilities as part of larger efforts to discover novel carbohydrate-active enzymes. Here, we announce the genome sequence of an S. marcescens strain (PIC3611) that is able to utilize complex polysaccharide substrates.

## ANNOUNCEMENT

**FIG 1:**
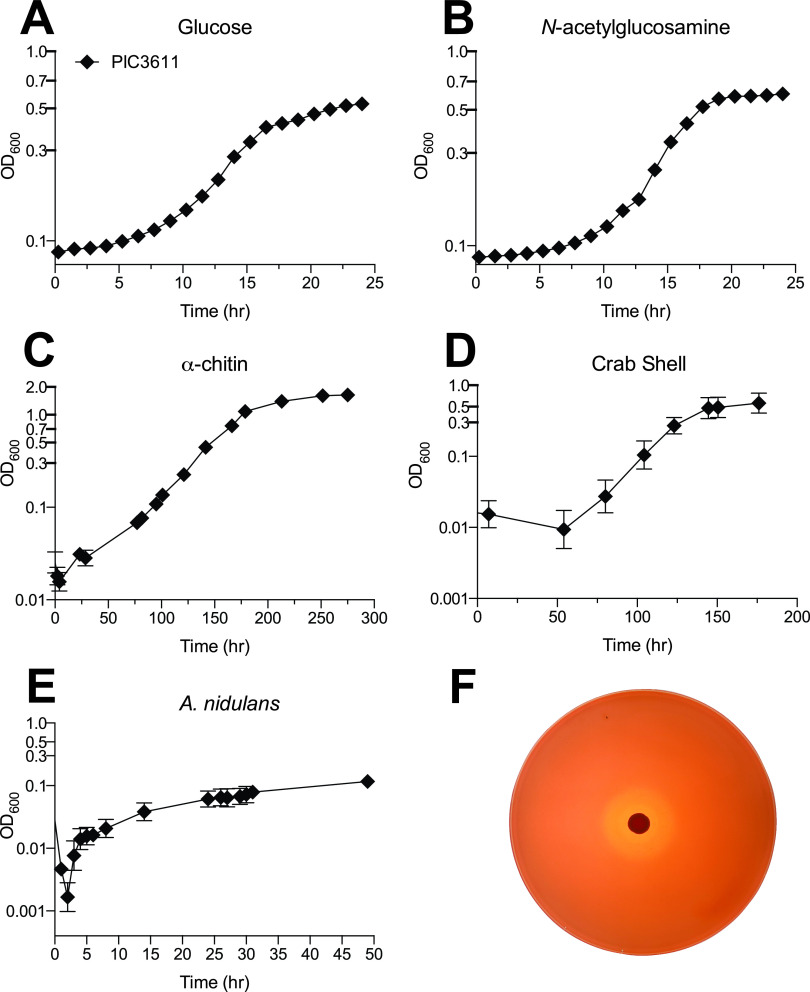
(A to E) Growth phenotypes of S. marcescens PIC3611 on 0.2% (wt/vol) glucose (A), 0.25% (wt/vol) *N*-acetylglucosamine (B), 1% (wt/vol) α-chitin (C), 5% (wt/vol) crab shell (Callinectes sapidus) (D), and 5% (wt/vol) fungal biomass (Aspergillus nidulans) (E) as the sole carbon sources. (F) Chitinase secretion of S. marcescens PIC3611 on 0.5% (wt/vol) colloidal chitin as the sole carbon source, as shown by Congo red staining. All media and plates were made with MOPS minimal medium and supplemented with the designated carbon source. All growth experiments were completed in biological triplicate, with error bars representing standard deviations, although some are too small to be observed. Growth analyses on glucose and *N-*acetylglucosamine were completed in an EPOCH2 microplate reader (BioTek), while growth on α-chitin, fungal biomass, and crab shell were measured using test tubes and a spectrophotometer (Milton Roy Spec20D+).

Serratia marcescens is a Gram-negative bacterium known for its red pigmentation and potent degradation of marine polysaccharides, particularly chitin (1–3). The S. marcescens strain PIC3611, which was previously available at Presque Isle Cultures (PIC), has a robust ability to degrade various complex chitin-containing substrates (Fig. 1). Despite the closure of PIC, S. marcescens strain PIC3611 is still used as a model system (4–6), which justifies genome sequencing.

S. marcescens PIC3611 was stored in 50% glycerol (wt/vol) at −80°C. The strain was grown to full density (optical density at 600 nm [OD_600_] of 1.5) in a morpholinepropanesulfonic acid (MOPS)-glucose (0.2% [wt/vol]) broth at 30°C for 48 h, and cell pellets were collected as described previously (7). The pellets were flash frozen in a dry ice-95% ethanol bath and then stored at −80°C before DNA extraction and whole-genome sequencing at Azenta (South Plainfield, NJ). Genomic DNA was extracted with a PureLink genomic DNA minikit (Invitrogen, Waltham, MA) according to the manufacturer’s instructions. Extracted DNA was quantified using a Qubit 2.0 fluorometer (Life Technologies, Carlsbad, CA). DNA library preparation used a NEBNext Ultra DNA library preparation kit (New England Biolabs, Inc., Ipswich, MA) according to the manufacturer’s instructions. The adaptor-ligated DNA library was cleaned and validated using a TapeStation system (Agilent, Santa Clara, CA) and quantified via a Qubit 2.0 fluorometer with real-time PCR (Applied Biosystems, Carlsbad, CA) analysis as needed. The DNA library was added to a single flow cell and sequenced on an MiSeq instrument (Illumina, San Diego, CA) using a 2 × 250-bp paired-end read configuration. For all software referenced below, default parameters were used unless otherwise specified. MiSeq Control Software (v2.6) was used for base calling. The raw sequence files (.bcl) generated by MiSeq sequencing were converted to FASTQ files and demultiplexed via Illumina bcl2fastq software (v2.17), allowing one mismatch during index sequence identification (7). Reads were trimmed using Trimmomatic (v0.36) with the following parameters: LEADING:3, TRAILING:3, SLIDINGWINDOW:4:15, and MINLEN:30 (8). *De novo* genome assembly was completed with the SPAdes *de novo* assembler (v3.10) with the following parameters: –k 21,33,55,77,99,127 and –careful (9); this produced 299 contigs, with an *N*_50_ value of 899,515 bp, an average Q score of 35.22, and a minimum length of 1,000 bp (smaller contigs were manually filtered out). There was a total of 62,571,120 reads from the MiSeq sequencing, which corresponds to ~2,800× genome coverage ((number of reads x 250 bp read length)/genome size). Sequencing and assembly found that the S. marcescens PIC3611 genome was 5,531,323 bp with an average G+C content of 59%, both of which were in agreement with other sequenced S. marcescens strains (10, 11). A nucleotide BLAST search (12) of the 16S rRNA gene returned S. marcescens strain JWCZ2 (GenBank Accession: CP055161.1) as the top hit, with an E value of 0.0 (100% coverage and 100% identity), as further confirmation. Quality assessment of the genome assembly used the getorf function in QUAST (v4.2) (13). Functional assessment used the NCBI Prokaryotic Genome Annotation Pipeline (PGAP) (v6.0) (14). PGAP found 5,429 genes, of which 5,246 coded for proteins, 103 tRNAs, 47 pseudogenes, 18 noncoding RNAs, and 15 rRNAs.

### Data availability.

The NCBI BioProject accession number for this genome is PRJNA802829, and raw data files can be obtained from the NCBI SRA under accession number SRX14024400. The genome sequence for S. marcescens PIC3611 can be found in the NCBI GenBank database under accession number JAKQYC000000000 and assembly number ASM2260299v1.
